# Antimicrobial resistance: a global view from the 2013 World Healthcare-Associated Infections Forum

**DOI:** 10.1186/2047-2994-2-31

**Published:** 2013-11-18

**Authors:** Angela Huttner, Stephan Harbarth, Jean Carlet, Sara Cosgrove, Herman Goossens, Alison Holmes, Vincent Jarlier, Andreas Voss, Didier Pittet

**Affiliations:** 1 Infection Control Programme and WHO Collaborating Centre on Patient Safety, University Hospitals of Geneva, Rue Gabrielle-Perret-Gentil 4, 1205, Geneva, Switzerland; 2 Fondation Hôpital St Joseph, Paris, France; 3 Division of Infectious Diseases, Johns Hopkins University School of Medicine, Baltimore, Maryland, USA; 4 Department of Medical Microbiology, Vaccine and Infectious Disease Institute, University of Antwerp, Wilrijk, Belgium; 5 Department of Infectious Diseases and Immunity, Imperial College London, The Centre for Infection Prevention and Management, London, UK; 6 Laboratory of Bacteriology-Hygiene, Assistance Publique-Hôpitaux de Paris, Hôpital Pitié-Salpêtrière, Université Pierre et Marie Curie-Paris 6, Paris, France; 7 Department of Medical Microbiology and Infection Control, Radboud University Nijmegen Medical Centre and Canisius-Wilhelmina Hospital, Nijmegen, The Netherlands

**Keywords:** Antimicrobial resistance, Antimicrobial conservation, Antibiotic stewardship, Infection control, Hand hygiene, Surveillance networks, Care bundles, Environment, Regulations, Human medicine, Animal medicine, Global health, World Healthcare-Associated Infections Forum

## Abstract

Antimicrobial resistance (AMR) is now a global threat. Its emergence rests on antimicrobial overuse in humans and food-producing animals; globalization and suboptimal infection control facilitate its spread. While aggressive measures in some countries have led to the containment of some resistant gram-positive organisms, extensively resistant gram-negative organisms such as carbapenem-resistant enterobacteriaceae and pan-resistant *Acinetobacter* spp. continue their rapid spread. Antimicrobial conservation/stewardship programs have seen some measure of success in reducing antimicrobial overuse in humans, but their reach is limited to acute-care settings in high-income countries. Outside the European Union, there is scant or no oversight of antimicrobial administration to food-producing animals, while evidence mounts that this administration leads directly to resistant human infections. Both horizontal and vertical infection control measures can interrupt transmission among humans, but many of these are costly and essentially limited to high-income countries as well. Novel antimicrobials are urgently needed; in recent decades pharmaceutical companies have largely abandoned antimicrobial discovery and development given their high costs and low yield. Against this backdrop, international and cross-disciplinary collaboration appears to be taking root in earnest, although specific strategies still need defining. Educational programs targeting both antimicrobial prescribers and consumers must be further developed and supported. The general public must continue to be made aware of the current scale of AMR’s threat, and must perceive antimicrobials as they are: a non-renewable and endangered resource.

## Background

There is nothing new under the sun, least of all antimicrobial resistance (AMR) [1]. Microbes that are antibiotic producers have always needed to be resistant to their own antibiotic. What is rapidly changing, however, is the scale of this resistance and its impact on human beings. Microbes have globalized along with their hosts, while at the same time antimicrobial consumption by these hosts—both humans and animals—has exploded. The gene pool for antimicrobial resistance has never been so accessible, nor its selection pressure so strong.

Warnings of increased resistance are not new. Some were issued prominently well before antimicrobials became widely available. Alexander Fleming’s Nobel Prize acceptance speech is often cited for his admonition that “it is not difficult to make microbes resistant to penicillin in the laboratory by exposing them to concentrations not sufficient to kill them…there is the danger that the ignorant man may easily under-dose himself and, by exposing his microbes to nonlethal quantities of the drug, make them resistant” [2].

**Figure 1:**
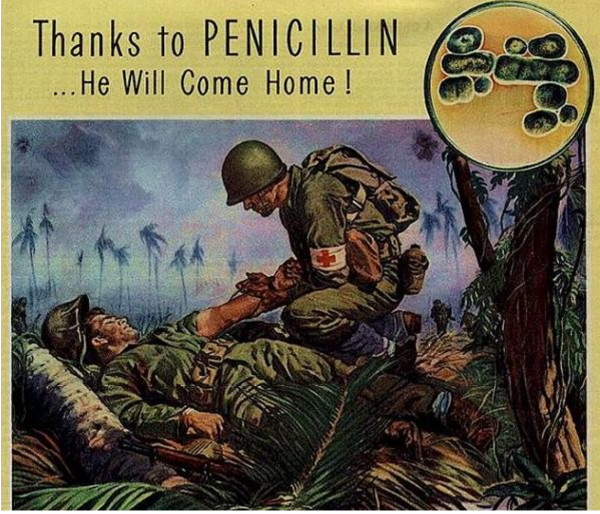
**An advertisement by Schenley Laboratories Inc.** By 1944, laboratories across the country were increasing penicillin production. Schenley’s advertisement stated, "When the thunderous battles of this war have subsided to pages of silent print in a history book, the greatest news event of World War II may well be the discovery and development of penicillin." Credit: Research and Development Division, Schenley Laboratories Inc., Lawrenceburg, Indiana, USA.

But while resistance in the targeted organism was a concern, the massive collateral resistance among the myriad “bystander” organisms composing the human microbiome was likely not anticipated [3]. Meanwhile, the miracle drugs were transformative, both in reality and in the psyche of millions (Figure 1). In 1954, the USA produced just under 1 million kilograms of antimicrobials; annual production in this country alone now exceeds 16 million kg [4]. The magic bullet described by Paul Ehrlich had launched, and its life-saving trajectory continues.

Our ability to develop and mass-produce over 25 classes of antimicrobials in seventy years may seem monumental—indeed, many hailed the new antibiotic era as the end of infectious diseases. Unfortunately, the game is in fact rigged. The infinitesimal generation time of a microbe will always confer it the advantage: it has infinitely more opportunities to gain resistance genes than we have to create new antimicrobials [5]. In this race, humans are being outrun: there have been no successful discoveries of new classes of antibiotics since 1987, while new, multi-resistant pathogens such as carbapenem-resistant enterobacteriaceae (CRE) are spreading with unprecedented alacrity [6].

The fourth biennial World Healthcare-Associated Infections Forum (WHAIF) met in June 2013 specifically to address the rapid spread of AMR. Seventy world experts from over thirty countries gathered in Annecy, France, to present and discuss their various successes—and failures—in the domain of AMR in order to better define future strategies to meet its challenge. In this position paper, we summarize the Forum’s key messages and conclusions.

### AMR’s increasing prevalence

**Figure 2:**
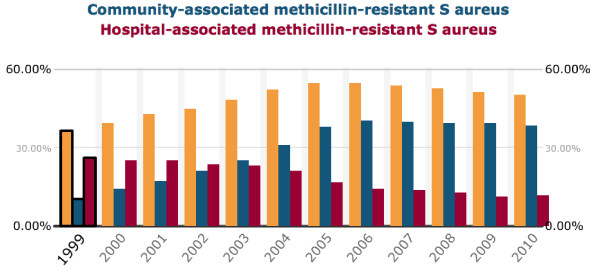
**Community-associated, hospital-associated, and overall MRSA prevalences in the United States from 1999 through 2010.** From the Center for Disease Dynamics, Economics and Policy (http://www.cddep.org).

Just three years after Fleming’s warning, 38% of *Staphylococcus aureus* strains in one London hospital were penicillin-resistant [7]. Currently, roughly 90% of strains in the UK [8] and nearly all of those in the US are resistant to penicillin, while in some communities more than 50% of strains are resistant to methicillin [9]. Resistance among gram-positive organisms is familiar territory. The arc of methicillin-resistant *S. aureus* (MRSA) has been well documented in Europe and the US, and the appropriate international reaction to its spread in the 2000s has allowed for a relative plateau in the prevalence of hospital-associated, if not yet community-associated, strains in these countries (Figure 2). Although still on the rise in some European and Asian countries [10,11], vancomycin-resistant enterococci (VRE) have also slowed their spread in North America in response to similarly targeted infection control measures [12].

In contrast, the new landscape of resistant gram-negative bacteria is unfamiliar and even more alarming. Organisms producing extended-spectrum beta-lactamases (ESBL) have increased their prevalence in Europe and elsewhere [13,14], and in some areas are “crossing the border” from hospital settings to the community. A recent prospective surveillance study of 1,713 asymptomatic, urban-dwelling volunteers in the Netherlands, frequently referred to as a country with “little” AMR, revealed the presence of ESBL-producing enterobacteriaceae in 8% of stool samples; [15] this prevalence is only slightly lower than that found in symptomatic patients of the same region (10.6%) [16].

The emergence and rapid spread of carbapenemase-producing gram-negatives such as extensively drug-resistant *Acinetobacter* spp. and enterobacteriacae producing New-Delhi metallo-protease-1 (NDM-1), *Klebsiella pneumoniae* carbapenemase (KPC), or oxacillinase 48 (OXA-48) are disturbing, as these multidrug-resistant infections leave patients with few or no antimicrobial options. In March 2013, the director of the US Centers for Disease Control and Prevention (CDC) called a press conference to report that these “nightmare bacteria," unseen in the country before 2001, had increased their prevalence fourfold in a mere decade. Invasive CRE infections carry mortality rates of 40 – 50% [17].

In the West at least, CRE appear to be largely confined at present to the hospital setting, but a jump to the community, as seen with MRSA, is a real prospect for the near term. A recent environmental point prevalence study conducted by Walsh and colleagues in New Delhi, India, revealed the presence of the NDM-1 gene in two of fifty community drinking water samples and twelve of 172 seepage samples [18]. Indeed, the obvious publication bias of data from the US and Europe likely creates a distorted view of the current overall prevalence of and mortality due to multi-resistant organisms. The burden of drug-resistant infections in low-income countries is of course difficult to quantify, but appears to be manifold higher than those in high-income countries [19,20].

Organisms that are almost entirely community-acquired such as *Neisseria gonorrhoeae* also continue to increase their resistance. Once sensitive to penicillin, tetracycline and fluoroquinolones, *N. gonorrhoeae* ’s current resistance rates leave only ceftriaxone as viable therapy in many regions [21]. Unfortunately, this treatment of last resort is unlikely to hold: the H041 *N. gonorrhoeae* strain, which carries high-level ceftriaxone and cefixime resistance, was detected in Japan in 2011; [22] its phenotypic homologue, F89, was isolated in France in 2012 [23].

The economic burden of AMR continues to climb. The annual societal cost-of-illness for AMR is considered to be roughly $55 billion for the US alone [24], though economists warn that this is likely an underestimate. Based loosely on the “incremental” cost related to the additional treatment of resistant over susceptible primary infection, this estimate masks the critical economic burden that arises when resistance leads to the loss of many of the advantages in medical care that antimicrobials have enabled [25]. It also excludes the indirect costs of resistance, such as prolonged morbidity, reduced productivity and related social effects. Studies of such broad scope have yet to be undertaken.

### Causes of antimicrobial resistance

The key promoter of antimicrobial resistance is the selection pressure caused by the use of antimicrobials. Overuse occurs across multiple ecosystems whose detailed analysis is beyond the present scope. Here we focus on antimicrobial consumption in humans and animals.

#### Selection and transmission of resistant pathogens in humans

Physicians and other healthcare workers have contributed to antimicrobial overuse through both omission and commission. It is difficult for clinicians to place concerns regarding the general concept of AMR before the immediate needs of the patient at point of care. Particularly in the outpatient setting, the assumption among healthcare workers that a patient expects an antimicrobial may be the most important driver of antimicrobial over-prescription [26].

Meanwhile, those who have taken a more active stand against AMR may have unwittingly exacerbated the problem. Until very recently, guidelines recommended unnecessarily long courses of antibiotics to avoid the development of resistance among targeted organisms: [27] Fleming’s warning was followed to the letter. But the antimicrobial is agnostic of its target; prolonging its administration prolongs the selection pressure favoring an evolution toward resistance among all affected microbes. More data are urgently needed on the optimal duration of antimicrobial therapy for various types of infection to better define instances where courses can be curtailed without placing the individual patient at risk of relapse.

Once resistance has been selected for, rapid human-to-human transmission of pathogens with resistant genes enables their spread. Although awareness is increasing, basic infection control measures such as hand hygiene are still performed suboptimally in many settings [28]. Isolation and cohorting measures are often delayed or altogether omitted by delays in diagnosis or their high relative cost, respectively [29]. Among other phenomena, globalization has enabled “medical tourism,” which facilitates in unprecedented fashion the international spread of resistance [30,31].

#### The role of antimicrobial consumption by food-producing animals

Much focus has been placed on human antimicrobial overconsumption as the main driver of AMR spread, likely because at present this is where the data are. But the consumption of antimicrobials by food-producing animals worldwide dwarfs that of humans, and the evidence base on their overconsumption as a powerful driver of AMR in humans and animals alike is growing swiftly.

The European Union’s collective ban on the non-therapeutic feeding of antibiotics of human importance to farm animals began in 2006 [32]. But other regions have not followed suit. The example from the US, a country with more than 300 million human inhabitants and over 10 billion food-producing animals, is particularly disheartening. After decades of little oversight of antimicrobial use in the agricultural industry, the Food and Drug Administration (FDA) finally released aggregate data in 2009 on sales of antimicrobials within the agricultural sector [4]. The discovery that a staggering 80% of all antimicrobials are destined for food-producing animals, and not humans, has prompted several calls for more aggressive surveillance of agricultural and veterinary practices [33,34].

Yet governmental response has been weak and industry resistance strong. Pharmaceutical and agricultural productions are not formally required to provide data on antibiotic sales and practices (the figures released in 2009 were based on voluntary reporting), making it impossible to verify claims of judicious use in animals. Furthermore, there is no agreement on definitions or measures of “standard” use. It is known that antimicrobials are systematically placed in animal feed at low levels for growth promotion, and typically at higher levels for “metaphylaxis,” which is a euphemism for over-treating large numbers of healthy animals.

The American example is playing out elsewhere. Countries in regions such as East Asia, Southeast Asia and South America are increasing their meat consumption exponentially, while most have not yet established mechanisms for the oversight of antimicrobial administration to animals, let alone bans on non-therapeutic applications.

Meanwhile, the evidence for the link between antimicrobial administration to animals and microbial resistance in humans is steadily increasing. In a 2011 study, a high prevalence of ESBL-coding genes was found in retail chicken meat (79.8%); genetic analysis showed that the predominant ESBL-coding genes in chicken meat and rectal swab specimens from humans of the same geographic region were identical. The same genes were also frequently found in human blood culture isolates [35]. Other data clearly show that antibiotic administration to chickens leads to more ESBL-producing enterobacteriaceae in chicken meat, [36] and that in humans such bacteria confer an increased risk of antibiotic failure and fatal infections [37]. A recent nested case-control study of more than 400,000 primary care patients from a single health care system in Pennsylvania from 2005 to 2010 revealed exposure to crop fields where swine manure was used as fertilizer as a significant risk factor for both community- and healthcare-associated MRSA strains [38], echoing previously reported data from the Netherlands [39].

### Steps forward: successful strategies

Several attempts have been made on local, regional and international levels to staunch the spread of AMR, essentially by (1) decreasing the human-to-human transmission of resistant pathogens, (2) decreasing the excessive consumption of antimicrobials, particularly those with broad-spectrum antimicrobial activity and, more recently, (3) facilitating the development of novel antimicrobials [40,41]. All measures have met with varying success. Table 1 summarizes abstracts presented at the 4^th^ WHAIF; these provide novel information on antimicrobial use and resistance, the emergence and control of endemic resistance, and antimicrobial conservation.

**Table 1:** Summary of abstracts presented at the 2013 World Healthcare-Associated Infections Forum

**I. Antimicrobial use and resistance**				
**Author**	**Short title**	**Study design**	**Setting**	**Key findings**
**Balkhy et al.**	Susceptibility of isolates from patients with VAP in Saudi Arabia	Retrospective susceptibility study	Single adult ICU, Saudi Arabia, 2004 – 2009	*• Acinetobacter* spp. was highly resistant (70 – 90%) to all tested antimicrobials including carbapenems (78% had four-class MDR)
				*• Klebsiella* spp. had low (0 – 14%) resistance with no detected MDR
**Carrel et al.**	MRSA and proximity to concentrated animal feeding operations	Retrospective unmatched case-control study	Veterans Affairs Hospital, Iowa, USA, 2009 – 2011	*•* High swine exposure (residential proximity to CAFOs) was associated with an increased risk of MRSA colonization
**Dantes et al.**	National burden of invasive MRSA infections, USA 2011	Prospective, population-based surveillance study	USA, 2011	*•* Compared to 2005, hospital-onset MRSA infections decreased by 54%, but community-associated infections remain stable
				*•* Invasive MRSA infections are now more common among persons in the community than hospitalized patients
**Datta et al.**	Quantifying MDRO exposure from patients in a single hospital to all California facilities	Retrospective case-cohort study	California hospitals and long-term-care facilities, 2005 – 2009	*•* Within a 5-year period, 1,198 patients with MRSA in a single hospital later exposed 137 hospitals and 103 LTCF
**Gastmeier et al.**	Dramatic increase of vancomycin-resistant enterococci in Germany with a belt of high proportions	Prospective surveillance study	> 600 ICUs and > 300 surgical wards throughout Germany, 2007 – 2012	*•* Healthcare-associated VRE colonization and infections are increasing dramatically in Germany, particularly SSI and primary BSI
				*•* There is a belt of significantly higher VRE proportions running through the middle of Germany
**Gikas et al.**	Antimicrobial use and HAI prevalence in Greek hospitals	Antimicrobial and HAI point prevalence study	37 hospitals, Greece, 2012	*•* 54.7% of hospitalized patients were receiving an antimicrobial (a slight increase from 51.4% ten years before)
				*•* ICU and surgery patients received the highest proportion of antimicrobials
				*•* 9% of patients had documentation of a HAI
**Gniadkowski et al.**	Obstacles in controlling KPC spread in Poland	Retrospective surveillance study	Poland, 2008 – June 2013	*•* KPC-producing enterobacteriaceae have spread rapidly throughout Poland since their emergence in 2008
				*•* Most common infection type is UTI; infections are most commonly reported by ICUs
**Hsueh PR**	Antimicrobial drug resistance in Asia Pacific	Prospective and retrospective surveillance studies	Taiwan, 2002 – 2011	*•* VRE infections in Taiwanese ICUs are increasing dramatically
				*•* ESBL-producing *Escherischia coli* infections have been increasing in Asia Pacific
				*•* Resistance to colistin (polymyxin B) is emerging in *A. baumannii* and *Pseudomonas aeruginosa*
**Kaku et al.**	Trends of antimicrobial resistance in a Japanese hospital	Retrospective surveillance study	Tertiary care hospital, Japan, 2012 – April 2013	*•* ESBL-producing bacteria are increasing in prevalence
				*•* Trends in MRSA and multi-resistant *Pseudomonas* are stable
**Mushtaq et al.**	Prevalence of carbapenemase carriage among inpatients in Karachi	Prospective surveillance study	Tertiary care hospital, Pakistan, 2012	*•* Of 469 patients sampled on admission by rectal swab, 36% were positive for *bla*_NDM_ and 92% for *bla*_CTX-M-15_
**Reuland et al.**	Risk factors for carriage of ESBL-producing enterobacteriaceae in the community	Prospective cohort study with nested, unmatched case-control study	Adult, community-dwelling volunteers, Netherlands, 2012	*•* Of 1713 stool samples from community-dwelling volunteers, 8% were positive for ESBL-producing enterobacteriaceae
				*•* Significant risk factors were hospital admission in a foreign country, antimicrobial use, and antacid use
				*•* ESBL-encoding genes CTX-M-15 and -14 were significantly associated with travel to Africa and the Middle and Far East, while CTX-M-1 had no association with travel
**Thu le et al.**	Antimicrobial use and resistance in surgical patients in Vietnam	Literature review	Vietnamese hospitals, 2010 – 2012	*•* Antimicrobials are prescribed inappropriately in Vietnamese surgical patients
				*•* The chief reason for prolonging antimicrobial therapy was the perception of a “poor environment”
**II. Emergence and control of endemic resistance**				
**Author**	**Short title**	**Study design**	**Setting**	**Key findings**
**Adler et al.**	Characteristics of an outbreak caused by OXA-48-producing CRE in a neonatal ICU in Jerusalem	Combined retrospective and prospective before-after cohort study	Neonatal ICU, Israel, 2012	*•* At the peak of the outbreak, one third of ICU patients acquired OPE
				*•* After the implementation of a bundled intervention, which included cohorting colonized patients, frequent rectal surveillance, and improving the implementation of infection control practices, no new cases were detected over the following three months
**Baltieri et al.**	Prevention of *Staphylococcus aureus* infection in the NICU: routine surveillance and decolonization	Combined retrospective and prospective before-after cohort study	Brazilian NICU, 2010 – 2012	*•* In response to increasing MRSA prevalence, universal NICU screening and decolonization (nasal mupirocin and oral hygiene with chlorhexidine for one week) were implemented
				*•* The fraction of MRSA infections decreased from 2% to 0.4% after bundle implementation; there was no significant impact on MSSA infections
				*•* There was no microorganism replacement phenomenon
**Cheung et al.**	Overcoming hand hygiene fatigue by involving the link nurses	Before-after study	Tertiary care hospital, Hong Kong, 2008 – 2012	*•* The activities of link nurses helped to increase compliance with hand hygiene practices from 50% to 83%
**Fournier et al.**	Emerging MDRO: same risk of outbreaks?	Prospective surveillance study	38 hospitals, France, 2010 – March 2013	*•* Incidence of secondary cases of VRE and CRE was significantly lower if cohorting and dedicated nursing staff and/or barrier precautions were employed within two days of detection of the index case
				*•* If these measures were delayed beyond two days, VRE spread was more significant than that of CRE
**Grall et al.**	Can the medical device DAV132 decrease the impact of antibiotics on fecal microbiota?	Experimental animal models (porcine, canine, murine)	France, 2013	*•* DAV132 is an oral medical device designed to deliver an adsorbent to the distal ileum that interferes with antibiotic absorption distal to the ileocecal junction
				*•* DAV132 captured gut antibiotic residues in dogs treated with intravenous levofloxacin without impacting blood pharmacokinetics and, in mice, significantly reduced the impact of cefotaxime on resistance to colonization by beta-lactam resistant enterobacteriaceae
**III. Antimicrobial conservation**				
**Author**	**Short title**	**Study design**	**Setting**	**Key findings**
**Awang Jalil et al.**	Infection prevention and control strategies of MDR infections in a neonatal ICU	Before-after study	Neonatal ICU, Malaysia, 2012 – April 2013	*•* An antimicrobial conservation program with dedicated staff was implemented, with resultant improvement in hand hygiene practices (95% compliance) and a sharp decline in HAI sepsis rates
**Bailin et al.**	Antimicrobial treatment for UTI among patients with total hip or knee arthroplasty	Two-stage combined retrospective and prospective cohort study	Tertiary care hospital, USA, 2011 – 2012	*•* Pre-operative screening for UTI was conducted in 95% of patients undergoing total hip or knee arthroplasty, regardless of symptoms; post-operative removal of the urinary catheter was also followed by urinalysis in 99% of patients regardless of symptoms
				*•* Nearly half (45.5%) of patients received antimicrobials pre- or post-operatively
				*•* In the prospective study, receipt of antimicrobials was not associated with signs and symptoms of UTI
**Bavestrello et al.**	Impact of intervention on antimicrobial consumption in aquaculture in Chile	Before-after economic analysis	Chile, 2008 – 2009	*•* After regulations on antimicrobial use in the salmon industry were introduced in late 2008, importation of fluoroquinolones decreased dramatically
**Edmunds et al.**	Assessing the need for antimicrobial use guidelines among staff of a Saudi Arabian hospital	Voluntary survey	Tertiary care hospital, Saudi Arabia, 2013	*•* Physicians’ responses to clinical vignettes in this survey showed good awareness of appropriate antibiotic options for various infections
				*•* Correct answers were not associated with age group, gender, or training status
**Glass-Kaastra et al.**	Variation in antimicrobial use patterns in Canadian Provinces	Retrospective population-level surveillance study	Canadian provinces, 2000 – 2010	*•* Although overall antimicrobial use is declining, patterns of use vary by province
				*•* Quebec had the lowest overall antimicrobial use, Newfoundland the highest
**Guzman-Blanco et al.**	Pan-American Health Organization guideline for treatment of infectious diseases in Latin America	International guideline	Latin America, 2013	*•* This guideline was recently updated to include recent surveillance data from Central and South America
**Koo et al.**	Appropriateness of continued use of empirical vancomycin	Retrospective cohort study	Tertiary care hospital, South Korea 2012	*•* Only 37.8% of systemic vancomycin prescriptions for 339 hospitalized patients over the year were deemed appropriate
**Ling et al.**	Case-control study to determine risk factors for CRE carriage	Retrospective matched (1:2) case-control study	Tertiary care hospital, Singapore, 2011 – 2013	*•* Significant risk factors were overseas hospitalizations in the past year, ICU admission, and exposure to carbapenems and fluoroquinolones
**Mehtar and Hara**	Antimicrobial stewardship in Africa-humble beginnings	Descriptive epidemiologic study	African hospitals, 2011 – 2012	*•* Only 14% of hospitals responding to a ESCMID survey reported having an ACP in place
				*•* The new Infection Control Africa Network is implementing ACP education programs in some African countries; long-distance learning and communication will employ mobile phone technology
**Moro et al.**	Impact of a regional intervention program to control carbapenemase-producing *Klebsiella pneumonia* (CPKP)	Before-after study	17 hospitals, Italy (Emilia-Romagna), 2011 – 2013	*•* Given the rapid spread of CRE in Italy, a bundled intervention targeting patients at increased risk for CRE was implemented in mid-2011 in the county of Emilia-Romagna
				*•* A significant deceleration in the spread of CRE was observed overall; in 5 hospitals the incidence rate of CPKP decreased from 32 to 15 cases/100,000 hospital patient-days
**Ndoye**	Antibiotic control in Senegal	Before-after study	Community hospitals, Senegal, 2008 – 2012	*•* Despite a national initiative issued in 2008 to establish site-based antibiotic conservation, regular assessments through 2012 reveal that nearly 60% of facilities have not begun any preparatory activity, and no facility has implemented recommended interventions fully
				*•* The chief reason appears to be a shortage of dedicated human resources
**Nicolle**	Antimicrobial stewardship in long-term-care facilities: what is effective?	Systematic literature review	Published studies retrieved through Medline & Embase, 1998 – 2013	*•* Engagement of internists and promoting infectious diseases consultations were very effective; strategies incorporating education, local guidelines, and feedback were less so
				*•* Specific programs to decrease UTI prophylaxis and treatment of asymptomatic bacteriuria were successful
**Nussenblatt et al.**	Inappropriate diagnosis and treatment of VAP is common in ICUs	Prospective observational study	ICUs in a single tertiary care center, USA, 2009 – 2010	*•* Antibiotics were continued for more than 3 days in patients without VAP (77%)
				*•* Those patients with inappropriately long antibiotic courses trended toward more symptomatic CDI and longer hospital stays
**Pulcini and Carlet for WAAAR**	A multidisciplinary initiative to save antibiotics: the World Alliance Against Antibiotic Resistance	Cross-disciplinary alliance	42 countries represented, 2011 –	*•* This alliance of nearly 500 individuals from 42 countries was formed in 2011
				*•* Non-profit organization composed of antimicrobial prescribers (physicians, veterinarians) and consumers, including politicians and environmentalists) open to all people worldwide
				*•* Goal is to decrease AMR’s prevalence
**Skov et al.**	Reducing antibiotic usage in Denmark: a campaign launch	Ongoing campaign	Danish general practitioners and general public, 2012 –	*•* In response to increasing AMR prevalence, a campaign to reduce unnecessary antimicrobial consumption has been launched
				*•* The campaign targets prescribers (physicians) and consumers (general public)

ACP: antimicrobial conservation program; AMR: antimicrobial resistance; BSI: bloodstream infections; CAFO: concentrated animal feeding operation; CDI: *Clostridium difficile* infection; CRE: carbapenem-resistant enterobacteriaceae; ESBL: extended-spectrum beta-lactamase; ESCMID: European Society of Clinical Microbiology and Infectious Diseases; HAI: healthcare-associated infection; ICU: intensive care unit; KPC: *Klebsiella pneumoniae* carbapenemase; LTCF: long-term-care facility; MDR: multi-drug resistant; MDRO: multidrug-resistant organism; MRSA: methicillin-resistant *Staphylococcus aureus*; MSSA: methicillin-susceptible *S. aureus*; NICU: neonatal intensive care unit; OPE: OXA-48-producing enterobacteriacae; SSI: surgical site infections; UTI: urinary tract infection; VAP: ventilator-associated pneumonia; VRE: vancomycin-resistant enterococci.

#### Strategies to reduce transmission

Reductions in human-to-human transmission of resistant organisms have been well documented via both horizontal and vertical measures [42]. Horizontal measures are not pathogen-specific and include interventions such as improved hand hygiene and enhanced environmental cleaning, both of which have been shown to interrupt pathogen transmission effectively [28,43,44]. Vertical measures are pathogen-specific and include targeted and universal screening upon hospital admission (e.g., MRSA, ESBL-producing gram-negatives) with or without preemptive isolation, often using novel molecular diagnostic techniques for rapid pathogen detection [45]. The decline in MRSA prevalence in many European countries over the last half-decade attests to the success of these approaches [41].

Newer strategies include the development of vaccines specifically targeting resistant organisms. OmpA and FepA, two immunogenic proteins found in ESBL-producing *K. pneumoniae*, were recently identified as promising vaccine gene candidates [46]. A vaccine composed of the outer membrane vesicles of *Acinetobacter baumanii* is currently being tested in animal models and appears to provide protection against at least some pan-resistant strains [47].

Other strategies are more indirect. The cytokine interleukin-12 (IL-12) is being tested as an “enhancer of adaptive immunity” in murine models infected with *N. gonorrhoeae*, which appears to suppress cell-mediated immune responses in the host. Local treatment of the mice with IL-12 generated gonococcus-specific immunoglobulin G and led to faster clearance of infection and induced resistance to reinfection, suggesting both a prophylactic and therapeutic role for IL-12 [48]. Should these newer approaches make it to the clinic, however, their long-term efficacy will be no more absolute than that of the antibiotics developed before them: the development of resistance is a dynamic process.

Finally, it should be noted that morbidity and mortality from infectious diseases were already declining sharply before the advent of antimicrobials and most vaccines: the implementation of sewage systems more than halved child mortality due to diarrheal illnesses by the early 20th century in several European countries [49]. This backbone of public health is now more critical than ever; effective sanitation systems worldwide are needed to decrease transmission of resistant microbes via wastewater.

#### Strategies to reduce consumption

Many hospitals employ antimicrobial conservation (AC) programs (traditionally known as antibiotic stewardship programs) to optimize both inpatient and ambulatory antimicrobial prescription practices. Several studies have documented an overall effectiveness of conservation programs with regard to this specific goal [50-54]. Much effort is now being expended to identify more specifically the obstacles to and facilitators of successful programs. Unfortunately, the reach of AC is limited. Worldwide, the vast majority of healthcare settings have not implemented AC programs. And in those countries with a relatively strong AC presence, most programs are found only in acute-care facilities, while evidence mounts that chronic-care facilities such as nursing homes and rehabilitation units, where prescription practices are largely unregulated, constitute equally important reservoirs of resistance [55]. And while AC can be linked to reductions in over-prescription, studies linking AC initiatives directly to reductions in AMR prevalence are still lacking.

Nonetheless, one clear benefit of system-wide AC programs is a subsequent reduction in symptomatic *Clostridium difficile* infections (CDI). Among other initiatives, the substantial decrease in fluoroquinolone and cephalosporin prescribing in the United Kingdom has led to a 70% reduction in symptomatic CDI over the past five years [56]. Indeed, CDI has become a driver for AC programs in some healthcare settings.

In the community, several landmark national campaigns appear to have achieved considerable success in reducing antimicrobial prescription. Campaigns attempt to educate not only healthcare professionals (antimicrobial prescribers), but also the general public (antimicrobial consumers) on the dangers of excessive antimicrobial usage; the latter appear to be particularly effective. France’s national campaign from 2002 to 2007 reduced the total number of antibiotic prescriptions per 100 inhabitants by 26.5% overall, with the greatest reduction (35.8%) in antibiotic consumers aged 6–15 years old [57]. Belgium’s national campaigns from 1999 to 2010 reduced the total number of antibiotic packages per 1000 inhabitants from 3.6 in 1999–2000 to 2.4 in 2009–2010 (a 33% reduction). *S. pneumoniae* resistance to penicillin decreased from 18% in 2000 to 7% in 2009. The total cost for reimbursement of antibiotics decreased by 21 million euros (−16.7%) from 125.5 million in 2002–03 to 104.5 million in 2008–09; cumulative savings in this period were thus 90 million euros (two thirds were due to reduced prescribing; one third was due to a reduction in the price of antibiotics). Because the costs of the six campaigns between 2002 and 2009 was 2.4 million euros, for every euro invested in the campaign, 25 were saved (H. Goossens, unpublished data).

And when a campaign is bolstered by concomitant governmental action, results can be impressive. In 1998, the Chilean Ministry of Health proactively enforced existing laws restricting the sale of antimicrobial agents without a medical prescription. These regulatory measures had a sustained impact on antimicrobial use in the outpatient setting: sales of oral agents decreased by nearly half within a few years [58].

**Figure 3:**
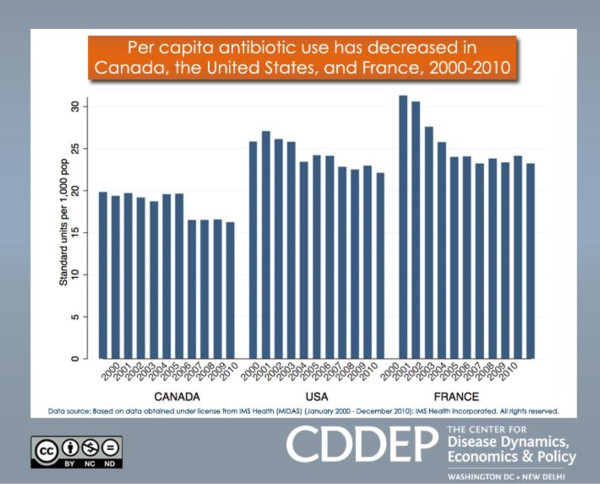
**A decline in human per capita outpatient antibiotic use in North America and France has been documented over the past decade.** From the Center for Disease Dynamics, Economics and Policy (http://www.cddep.org).

What is yet unclear, however, is the durability of such measures; a rebound rise in antimicrobial prescription may occur once a campaign, or a strict enforcement of existing regulations, is brought to a close. For the moment, at least, outpatient antimicrobial usage in North American, European, and some other countries appears to be on a modest decline (Figure 3).

As with AMR prevalence data, however, the view from Europe and North America may provide an overly sanguine impression of reduction rates in both antimicrobial consumption and resistance transmission. Antimicrobial conservation programs and awareness campaigns are essentially non-existent in many developing nations, where antimicrobial consumption data are lacking though, paradoxically, easy access to mass-produced, sometimes diluted (subtherapeutic) antimicrobials often is not.

Efforts to reduce antimicrobial consumption in food-producing animals date from as early as 1969, with Swann’s recommendation calling for a ban on the nontherapeutic use of antimicrobials in animals and agriculture in the United Kingdom [59]. Such a ban was highly contentious then, as now, and obstacles to its implementation are abundant in numerous countries.

#### Facilitating the development of novel antibiotics

Efforts are under way in some Western nations to fast-track the development of desperately needed novel antimicrobials. The research and development required to bring one antimicrobial successfully to market can exceed 1 billion, thus many in the pharmaceutical industry have prioritized drugs with a wider market and indications for long-term usage. A few public-private partnerships have been established, among them the Innovative Medicines Initiative (IMI), a joint undertaking between the European Commission and the pharmaceutical industry. The IMI acts as a neutral third party by supporting collaboration among experts from industry and academia, and seeks to accelerate antimicrobial discovery and development specifically by exploring effective incentives via economic models. In the US, the GAIN (Generating Antibiotic Incentives Now) Act went into effect in late 2012; it earmarks novel antibiotics for priority FDA review and mandates the creation of a pathogen-focused (rather than anatomic infection site-focused) antibacterial drug-development pathway, thus attempting to reduce financial impediments to drug development.

### Beyond the clinic

Globalization and current widespread agricultural practices prevent hospitals and patients from existing in a secure void. These external realities can quickly upend the benefits accrued by years of methodical AC and infection control measures in any one hospital, as demonstrated by the events following the admission of a sole patient harboring a carbapenemase gene to a US hospital [60].

Clinical interventions remain crucial, but they cannot suffice in the face of AMR’s current magnitude and cross-disciplinary complexity. Wide-ranging and highly coordinated efforts on several societal levels are imperative to slow the course of AMR. Policies requiring the tracking of antimicrobial dispensing in humans and animals are urgently needed. Data sharing and harmonization across national borders and industrial sectors, including pharma and biotechnology, are essential. Table 2 lists the ten most urgent priorities for action cited by participants of the 4^th^ WHAI Forum; these address policy-makers, industry leaders, and researchers alike.

**Table 2:** **The ten most urgent priorities for action against the spread of antimicrobial resistance cited by participants of the 4**^**th **^**WHAI Forum**

*For policy-makers and health authorities:*	
1	Limit the use of antimicrobials in food-producing animals by banning non-therapeutic applications, including growth promotion and metaphylaxis
2	Establish and enforce regulations on sales of antimicrobials for use in human medicine, including prohibition of over-the-counter sales worldwide
3	Develop a detailed charter on antimicrobial conservation to be ratified and upheld by ministries of health worldwide
4	Develop coordinated and culturally sensitive awareness campaigns targeting the general public and imparting the importance of protecting antimicrobials as a limited and non-renewable resource
5	Rigorously support the improvement of sanitation systems to eliminate resistant microbes in wastewater; regularly provide education about fundamental practices such as hand hygiene to prevent the spread of infection
6	Together with the pharmaceutical industry, explore (1) incentives to stimulate research and fast-track development of novel antimicrobials and (2) new economic models that reconcile public health interests with industry profitability
*For the human and veterinary healthcare communities:*	
7	Establish standardized, universal methods and metrics for surveillance of antimicrobial use and resistance development, respectively
8	In medical and veterinary school curricula, require universal and detailed instruction in microbial resistance development and the prudent use of antimicrobials; for physicians and veterinarians in training, require on-the-job refresher courses
*For the general public:*	
9	Include patients and other antimicrobial consumers in the development and implementation of action plans
*For industry:*	
10	Continue to develop and advance point-of-care rapid diagnostic tests to avoid the prescription of antibiotics for viral infections and allow more targeted therapy

Such measures will allow researchers and policy-makers to define more clearly the global clinical burden of AMR. This burden needs precise definition for several reasons, among them: (1) society’s finite resources for confronting AMR will be utilized more efficiently if they can be directed where most needed, (2) such “hard” data would swiftly repudiate the claim from certain industrial sectors that regulation of antimicrobial administration to food-producing animals and other like-minded measures are unnecessary because AMR’s present clinical impact is limited, (3) a growing awareness of the true dimensions of AMR’s global burden may effect a genuine paradigm shift [61] in society’s collective consciousness, such that antimicrobials may finally be viewed by the majority (and not simply by a small group of healthcare workers) as a non-renewable and highly endangered resource, thus placing AMR in a context of sustainable development.

Though the most abstract, this third hypothetical consequence could prove to be one of the most powerful elements of a future impact on AMR’s course. Currently, a near tragedy of the commons is playing out: individuals who benefit from a shared resource are effecting the rapid depletion of that resource through independent and rational action according to individual self-interest [62]. But most individuals do not yet perceive antimicrobials as a limited resource. It is hoped that if and when they do on a mass level, they will cooperate to support implementation of measures protecting the resource and thus the larger group’s long-term best interests.

By definition a paradigm shift is not reversible; indeed, any global perception of antimicrobials as a shared and endangered resource must be enduring. Evidence from the laboratory does not support the hypothesis that all microbes must pay a physiologic price for their acquired resistance, rendering them less “fit” than their wild-type counterparts [63]. That is, multi-resistant pathogens are here to stay, whether we achieve temporary or even lasting reductions in antimicrobial overuse or not.

#### An urgent need for international collaboration

There are indications that this awareness may be taking firmer root, and perhaps in places where it is currently most crucial. Though the Indian government’s immediate reaction to the publication of the Walsh study in 2011 was to forbid the transfer abroad of any additional Indian environmental specimens, this mandate has since been reversed, and official governmental support has been extended to the Indian medical societies’ recent Chennai Declaration, a call to action against the further spread of AMR [64].

Because of the tireless efforts of practitioners and researchers from both non-governmental (e.g., the Center for Disease Dynamics, Economics and Policy; the Alliance for the Prudent Use of Antibiotics; and the World Alliance Against Antibiotic Resistance) and governmental organizations (e.g., the United Kingdom’s Chief Medical Officer, Dame Sally Davies), an awareness of the scope of AMR is beginning to reach non-clinical and non-academic sectors. In 2012, Margaret Chan, Director General of the World Health Organization declared publicly that AMR could bring about “the end of modern medicine as we know it.” [65] The American CDC recently released its first-ever report on AMR, entitled “Antibiotic Resistance Threats in the United States, 2013”; in his foreword, director Thomas Frieden identified AMR as one of the nation’s most serious health threats [66].

These calls to action should, at least theoretically, accelerate the international collaboration urgently needed to confront a global menace. Such may be happening, albeit in belated fashion. At the G8 Summit in June 2013, science ministers identified AMR as the “major health security challenge of the 21^st^ century” and pledged intensive international collaboration to achieve the concrete goals of (1) avoiding the misuse of remaining antimicrobials and (2) streamlining and facilitating the development of new antimicrobials as well as (3) rapid diagnostics to accelerate the identification and treatment of resistant pathogens [67]. The formulation of such specific targets is encouraging: in recent decades the international community has been long on general rhetoric and short on coordinated, precise action.

## Conclusion

Antimicrobial resistance is a clear and present danger. Immediate and coordinated measures must be taken worldwide to safeguard remaining antimicrobials and facilitate the development of novel antimicrobials. Bans on nontherapeutic antimicrobial consumption in livestock must be effectively championed despite strong resistance from industrial sectors. Conservation programs must be further optimized and implemented in other, non-acute healthcare settings such as long-term-care facilities. Educational programs targeting both antimicrobial prescribers and consumers must be further developed and supported. The general public must continue to be made aware of the current scale of AMR’s threat. International collaboration among researchers and policy-makers must solidify to effect lasting reductions in the spread of antimicrobial resistance.

## Competing interest

The content of this paper expresses the views of its authors and in no way represents the position of their affiliations.

## Authors’ contributions

AHr conceived and drafted the manuscript and produced the different versions. DP and SH provided an extensive review of the first draft of the manuscript. SH, JC, SC, HG, VJ, AHs, and DP reviewed the manuscript and provided important intellectual content. All authors have read and approved the final version of the manuscript.
